# An automated photo-isomerisation and kinetics characterisation system for molecular photoswitches†

**DOI:** 10.1039/d5dd00031a

**Published:** 2025-06-30

**Authors:** Jacob Lynge Elholm, Paulius Baronas, Paul A. Gueben, Victoria Gneiting, Helen Hölzel, Kasper Moth-Poulsen

**Affiliations:** a Department of Chemical Engineering, Universitat Politècnica de Catalunya, EEBE Eduard Maristany 10-14 08019 Barcelona Spain kasper.moth.poulsen@upc.edu; b ICREA Passeig Lluís Companys 23 08010 Barcelona Spain; c Justus-Liebig-Universität Giessen, Institut Organische Chemie Heinrich-Buff-Ring 17 35392 Gießen Germany; d Department of Chemistry and Chemical Engineering, Chalmers University of Technology Gothenburg Sweden; e Institute of Materials Science of Barcelona, ICMAB-CSIC Bellaterra Spain

## Abstract

Physical chemistry parameters such as absorbance, photoconversion quantum yield, and thermal half-lives are crucial for the characterisation of new molecular photoswitch systems. In a traditional workflow, these parameters are challenging and time-consuming to measure. In this paper, a high-throughput flow-based photoswitch characterisation platform with a built-in broad-spectrum LED array and thermal back-conversion capabilities is developed with UV-Vis spectroscopic analysis tools to reduce materials consumption, limit laborous workflows, and improve experimental reproducibility. Following the experiments, an in-house developed Python program is used for easy and fast data analysis. The program is designed to be able to analyse different types of photoswitches depending on the molecular properties. The specific components and configurations are detailed, enabling reproducibility and adaptation to various experimental needs. This system demonstrates the potential for efficient, high-throughput analysis in spectroscopic studies. Wide applicability is underlined by showing the results and comparison of three different photoswitch types, norbornadienes, bicyclooctadienes, and azobenzenes. The results we obtain are in good agreement with reported values in the literature.

## Abstract 2

Physical chemistry parameters such as absorbance, photoconversion quantum yield, and thermal half-lives are crucial for the characterisation of new molecular photoswitch systems.

## Graphical abstract

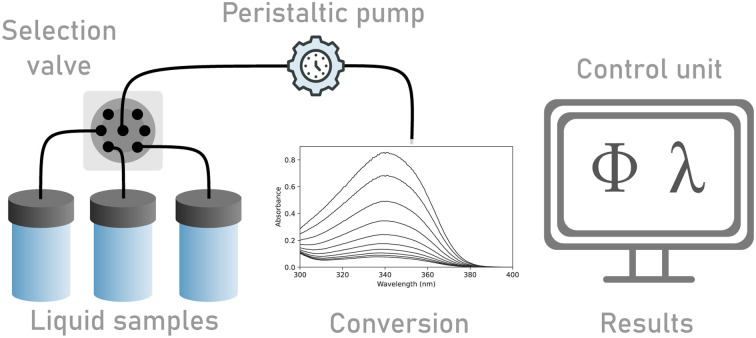

## 1 Introduction

The field of molecular photoswitches has garnered significant attention due to their potential applications in energy storage, molecular machines, and responsive materials.^1–3^ Photoswitchable molecules, such as norbornadiene (NBD),^4–6^ bicyclooctadiene (BOD),^7–9^ and azobenzene derivatives,^10,11^ exhibit reversible structural transformations between isomers under the influence of light or thermal stimuli. These isomerisations often result in distinct changes in physical and chemical properties, making them highly attractive for photochemical applications. However, understanding the kinetics of these processes, specifically photoconversion and thermal back-conversion rates, is critical for optimizing their performance and tailoring their behaviour for specific applications.^12^ Traditional methods for characterizing photoisomerisation and thermal relaxation processes often involve labour-intensive and time-consuming manual measurements.^13^ These methods can introduce variability and inefficiency, particularly when studying a range of photoswitches or exploring the influence of diverse environmental conditions such as solvent polarity, temperature, or wavelength of incident light. Automated setups designed for high-precision, reproducible kinetic studies are thus essential for advancing the systematic characterisation of these systems.^14–16^ Furthermore, high-throughput experimentation is essential in generating large datasets required to train robust machine learning algorithms.^17^ Here, we present a fully automated photoisomerisation and kinetics characterization setup designed to quantitatively study key physical properties of molecular photoswitches. The system integrates high-precision spectroscopic measurements with programmable light sources, valves, and pumps, leading to a system that enables simultaneous monitoring of both photoconversion and thermal back-conversion rates. The setup is optimised for high-throughput investigations and provides real-time data that allows for monitoring the behaviour of the photoswitches. The design allows for precise control of the experimental parameters, including light intensity, wavelength, and temperature, ensuring accurate and reproducible results across diverse molecular systems. We demonstrate the utility of the automated system by characterizing the photoisomerisation kinetics and thermal back-conversion of three representative photoswitch classes: norbornadiene–quadricyclane (NBD–QC), bicyclooctadiene–tetracyclooctane (BOD–TCO), and the *trans*–*cis* isomerisation of azobenzene. These molecules have been selected for their relevance in energy storage (NBD and BOD) and molecular machine applications (azobenzene). Employing this automated platform, we provide detailed insights into the kinetic behaviour of these systems, highlighting the role of structural features and local environmental factors in influencing their isomerisation dynamics. The data is analysed using an in-house developed Python program with broad and generalised applicability. The results underscore the potential of this setup to accelerate the development and optimization of molecular photoswitches for advanced technological applications.

## 2 Method

### 2.1 Sample preparation

The norbornadiene (2-cyano-3-(3,4-dimethoxyphenyl)-norbornadiene) used in these experiments was synthesised according to the literature^18^ and presented in Section S3.1 in the ESI.† For this sample, 1.2 mg of solid compound was dissolved in 100 mL of acetonitrile, yielding a concentration of 4.74 × 10^−4^ M. The bicyclooctadiene (ethyl-3-(2-methoxyphenyl)-bicyclo[2.2.2]-octa-2,5-diene-2-carboxylate) used in these experiments was synthesised according to literature^7^ and presented in Section S3.2 in the ESI.† The sample was prepared by dissolving 9.5 mg of solid compound in 100 mL of acetonitrile, leading to a concentration of 3.5 × 10^−4^ M. 5 mL of this solution was mixed with 5 mL of acetonitrile to obtain a final concentration of 1.75 × 10^−4^ M. The azobenzene used in these experiments was purchased from Fisher Scientific under the name Azobenzene, 97+% purity, Thermo Scientific Chemicals (CAS: 103-33-3). 8 mg of azobenzene was dissolved in 100 mL of acetonitrile yielding a concentration of 4.39 × 10^−4^ M. 1 mL of this solution was mixed with 9 mL of acetonitrile in a 20 mL vial resulting in a concentration of 4.39 × 10^−5^ M. The three samples were prepared in vials with membrane caps to allow easy connection to the automated platform.

### 2.2 Photoconversion

The photoconversion quantum yield is estimated using the formulation summarised by Börjesson *et al.*^13^ The general equation for most photoswitches is presented here:1
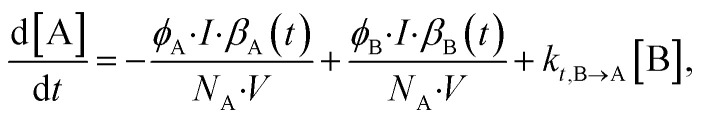
where [A] is the concentration of the reactant, [B] is the concentration of the photo-product, *ϕ* is the photoconversion quantum yield, *I* is the photon flux, *β*(*t*) is the time-dependent absorbance of each species, *N*_A_ is Avogadro’s number, *V* is the volume and *k* is the thermal back-conversion rate constant. The first term describes the forward photoconversion, the second term describes the backward photoconversion, and the third term describes the thermal back-conversion. This differential equation can be quite tedious to solve and fit to collected data, therefore, in certain cases, some approximations can be made. In the case of a photoswitch pair with a long thermal half-life (*i.e.* low *k* value) of at least 100 times the duration of irradiation, one can omit the last term regarding the thermal back-conversion to simplify the equation. In the case of zero or minimal spectral overlap between the photoproduct and the irradiation source, the term regarding the backward photoconversion can be omitted. In the high concentration regime, it is approximated that all photons are absorbed, which means that the term *β*_A_(*t*) is now equal to 1. However, in the low absorption regime, one needs to consider that the absorbance will change whenever the concentration changes. This makes it more complicated computational wise, due to the changes in the time-dependent absorbance, but it allows for performing the experiments with small amounts of sample due to the low concentrations (normally between 10^−3^ and 10^−5^ M at 10 mm optical path length). Furthermore, the conversion at this concentration can readily be monitored with UV-Vis spectroscopy. In all these situations, a monochromatic light source is assumed. To account for this, since the LED is not entirely monochromatic, one can integrate over the area of the LED and the absorbance of the compound to determine the number of photons absorbed following the method reported by Volfova *et al.*^19^ However, as recently reported by Volker *et al.*,^14^ this correction in practice, typically has minimal impact on their results.

### 2.3 Actinometry

A popular way to estimate the photon flux of a light source is to perform actinometry using ferrioxalate (iron(iii)oxalate) as the photochemical actinometer.^20–23^ This can be performed in many different setups, and the range of the functional wavelengths spans from around 200 nm to 600 nm, making it one of the most versatile actinometers. However, calibration of photon flux using ferrioxalate can be tedious in the preparation of the sample and the conversion of the ferrioxalate, and the data fitting afterwards. In this work, we measured the power of the LED with a powermeter connected to a photodiode. The measured power combined with the spectral shape of the LED readily yielded the photon flux by using the following equation:2
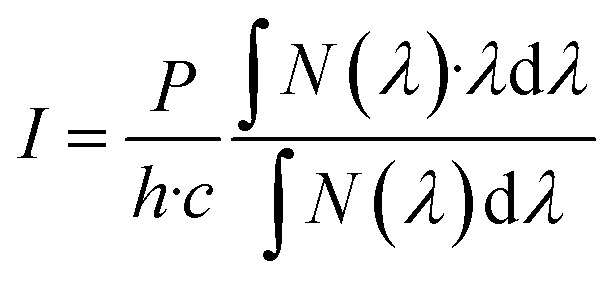
where *N* is the number of photons measured at the wavelength in the emission spectrum, *P* is the power measured by the powermeter, *h* is Planck’s constant, *λ* is the wavelength, and *c* is the speed of light. In comparison, the measurement of the photon flux with the powermeter takes approximately one second, whereas the determination of the photon flux with the ferrioxalate can take up to 30 minutes.

### 2.4 Thermal kinetics

The conversion in eqn (3) shows the reaction scheme of a T-type photoswitch, which is defined by having a meta-stable photoproduct that can spontaneously relax to the stable isomer under ambient conditions.3
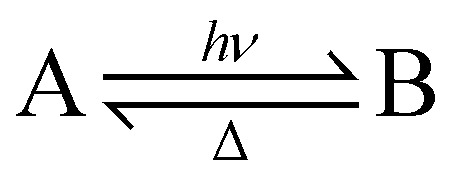


To estimate the thermal conversion kinetics, a simple first order reaction equation is used:

4 [A] = −[A]_0_·e^−*k*·*t*^ + *b*.

This equation works at a wavelength where the photoproduct, B, does not absorb in order to ensure a linear correlation between the absorbance and the concentration of the stable isomer. By fitting this equation to the obtained data, one can calculate the rate constant *k* at a specific temperature. These rate constants from at least three different temperatures can yield the thermodynamic properties of the photoswitch, which is shown in the Arrhenius and Eyring plots. The Arrhenius plot follows the equation:5
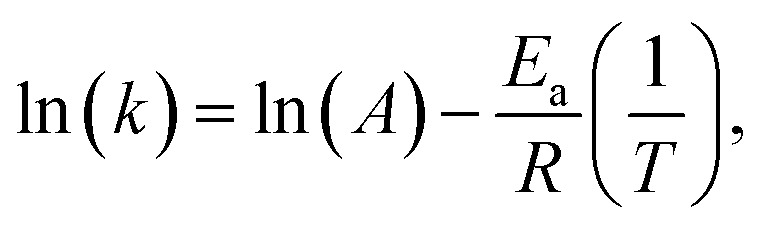
then a linear fit will yield ln(*A*) as the *y*-intersection and 
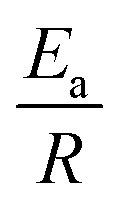
 as the slope, where *A* is the pre-exponential factor, *E*_a_ is the activation energy, and *R* is the ideal gas constant. In Fig. S1 in the ESI,† a comparison is shown of the different rate constants obtained according to how long the experiment is allowed to run.

### 2.5 Automation system

**Fig. 1:**
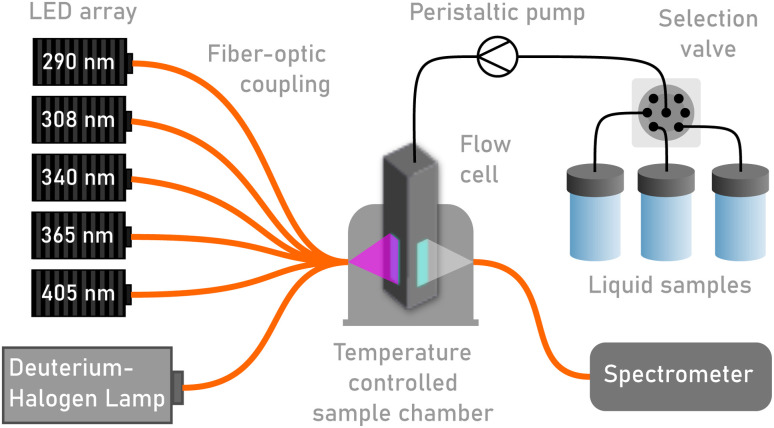
Schematic of the photoswitch characteristation platform.

The automated system is based on a flow system with several components, shown in Fig. 1. In the centre of the schematic is the UV-Vis flow cuvette placed in a temperature-controlled cuvette holder. On one side, connected *via* fibre optics, is a deuterium-halogen lamp and an array of LEDs. On the other side is a spectrometer also connected with fibre optics. In between the lamp and the cuvette is a collimating lens, and between the cuvette and the spectrometer is a focusing lens for optimal light collection to the spectrometer. A precision peristaltic pump, designed to minimise pulsations, is used to pump the liquid sample through the flow cuvette and into either a collection or a waste container. A selection valve is placed before the pump to select which sample should fill the flow cell. This also allows for fast switching to other solvents for cleaning procedures.

Since the window on the cuvette is relatively small (2 × 4 mm), the position of the cuvette within the cuvette holder must be very precise, otherwise movement of the cuvette can change the illumination path, thus leading to changes in the absorbance spectrum. This is accomplished by inserting small metal plates (0.1 mm) beside the flow cuvette in the sample holder to limit loose movement. The specific component models and manufacturers are provided in the ESI.† One challenge with the flow cuvette is that the sample within the sample chamber cannot be stirred. This means that the entire volume of the sample must be irradiated by the light source such that stirring is not necessary. To make sure of this, the irradiation area before and after the sample chamber was evaluated. The irradiation area before the sample chamber was determined to be sufficiently larger than the area of the window to the sample chamber. In contrast to that, the advantage of the flow cell combined with this setup is that each conversion experiment can be accomplished with less than 200 μL of new sample, and instead of waiting for back-conversion, one can simply flow 200 μL of sample through the tubing to commence a new conversion. For accessibility and interchangeability, the setup can also be used with normal cuvettes. It must be noted that using a normal cuvette will require stirring to ensure mixing during conversion. A disadvantage of using the normal cuvette is that at least 2 mL of sample is required for each experiment, unless the sample is back-converted between experiments. Additionally, a Python graphical user interface was developed for easy and highly customisable control of experiment parameters and to perform the experiment.

### 2.6 Optimised experimental workflow

In order to perform the experiment one must prepare the sample solution and place it in one of the positions available to the selection valve. If a different solvent than the pre-positioned ones is used, then a solvent vial must be placed in the solvent position. From here on everything will be controlled from the computer using the in-house graphical user interface (GUI) shown in Fig. S22 in the ESI.† First, the solvent will be pumped from the solvent vial through the electronically controlled valve, then through the pump and finally into the flow cuvette. From here, a reference spectrum will be taken, as required for measuring absorbance spectra, since the setup does not contain a double-beam spectrometer. To prepare the measurement, the sample is pumped into the flow cuvette by switching the valve and starting the pump. Then, the desired irradiation wavelength is chosen from a list of LEDs and the corresponding current of the LED driver is set. Once the desired UV-Vis measurement interval is set, the experiment can be started. The program will automatically turn “on” and “off” the LED in between measurements and open and close the lamp shutter when the UV-Vis spectrum is measured. The GUI shows a full spectrum and the time evolution of several chosen wavelengths for easy and continuous monitoring. The experiment will finish once the target is reached, which can be set to a specific time or a specific absorbance at a specific wavelength. Once the experiment is finished, a new one can be prepared by pumping around 200 μL of sample through the tubing. After all the experiments are finished, one can switch to the solvent valve and pump solvent until clean.

## 3 Results and discussion

The automated system can, for typical molecular systems, record the photoconversion quantum yield and back-conversion kinetics in less than 24 hours. By determining the thermal rate constant at three different temperatures, we obtain the thermodynamic parameters of the thermal back-reaction such as Δ*S*, and Δ*H* through Eyring analysis. Here we present a comparison of results between reported workflows and the newly developed automated setup. We analysed three different types of photoswitches with different molecular properties for the benchmark, a norbornadiene,^18^ an azobenzene,^24^ and a bicyclooctadiene.^7^ Although the experiment is designed to minimise the exposure of sample to the irradiation from the lamp, it is impossible to completely avoid it. In Fig. S10 in the ESI† we show the effect of the lamp exposure on the norbornadiene (NBD) sample. After 10 minutes of continuous UV-lamp irradiation of the compound, we see a conversion of roughly 10%. In the experiments, each spectrum is measured in less than half a second, which would translate to less than 0.01% conversion. With this information, we believe that the inclusion of lamp irradiation in the quantum yield measurements is negligible. Normally, the procedure of determining thermal half-lives is performed by converting the sample in a regular UV-Vis cuvette outside of the measurement instrument with an external LED. After the desired conversion is reached, the sample is placed in the UV-Vis instrument for periodic measurement of the absorbance. With this approach, the sample is only heated after the irradiation, which can cause inaccurate measurements until thermal equilibration. A small pressure build-up can also interrupt the spectrum measurements with leakage and spontaneous evaporation, causing changes in concentration. These obstacles are overcome in our experimental setup thanks to the small sample volume that requires heating.

### 3.1 Norbornadiene

**Fig. 2:**
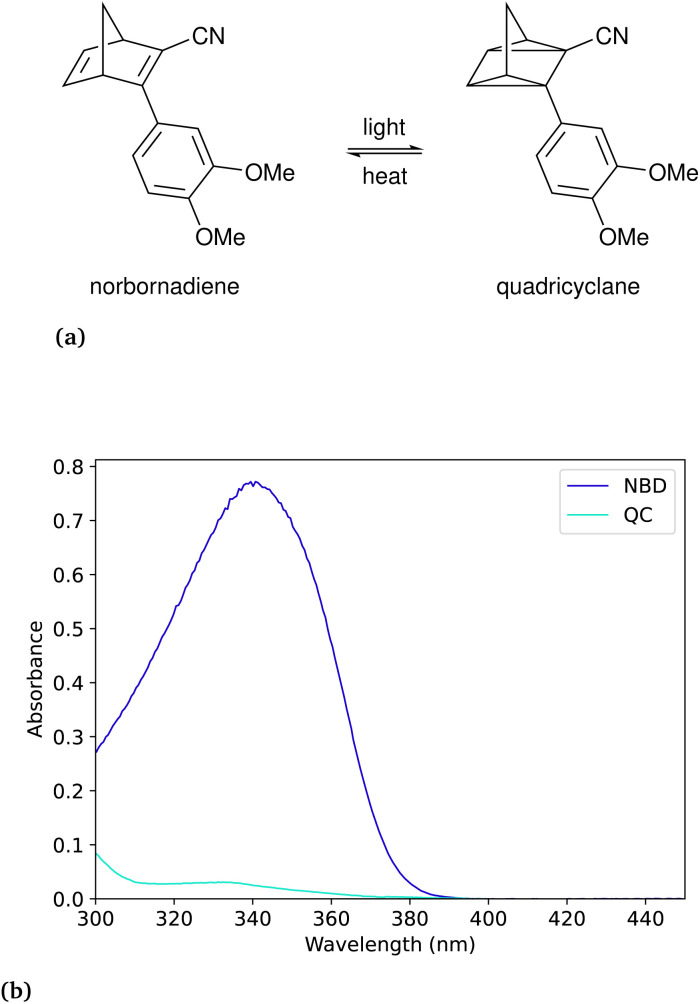
(a) Reaction scheme for the norbornadiene–quadricyclane (NBD–QC) photoswitch pair. (b) Corresponding UV-Vis spectra of the photoswitch pair.

The norbornadiene–quadricyclane (NBD–QC) pair shown in Fig. 2 has been published by Jevric *et al.*^18^ (2018) and the name of the compound is 2-cyano-3-(3,4-dimethoxyphenyl)norbornadiene. In the following part, it is simply referred to as the NBD, since we have not included other photoswitches of the same type. In the case of this pair, the UV-Vis spectrum of the NBD has a minimal overlap with the spectrum of the QC, as shown in Fig. 2(b). This allows for a simplification of eqn (1) to eliminate the term regarding the photo-induced back-conversion (*e.g.* the conversion from QC to NBD). In addition to this, the thermal half-life at room temperature is 30 days, which is magnitudes slower than the photoconversion time, due to a high photon flux and a low concentration, which allows for disregarding the term describing the thermal back-conversion from eqn (1) as well. An illustration of the difference is shown in Fig. S9 in the ESI.† This results in a simplified equation for the photoconversion:6
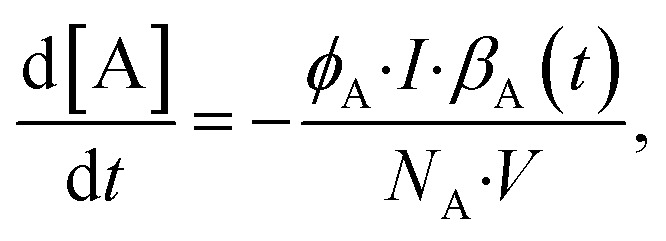
where only the first term regarding the forward photo-conversion is considered. Under the approximation that the light source is mono-chromatic, this differential equation has an analytical solution.

Additionally, we measured the thermal half-life of the corresponding QC. The thermal half-life of the NBD–QC pair was determined to be 27 days at 25 °C. This is in reasonable agreement with the thermal half-life of 28.7 days reported by Jevric *et al.*^18^ (Table 1).

**Table 1:** The determined quantum yield of the NBD at two different wavelengths of irradiation. The measurements were performed at 25 °C in toluene

Wavelength of irradiation	Number of measurements	This work	Jevric *et al.*^18^ (2018)
340 nm	7	0.676 ± 0.028	
365 nm	2	0.689 ± 0.022	0.68

### 3.2 Bicyclooctadiene

**Fig. 3:**
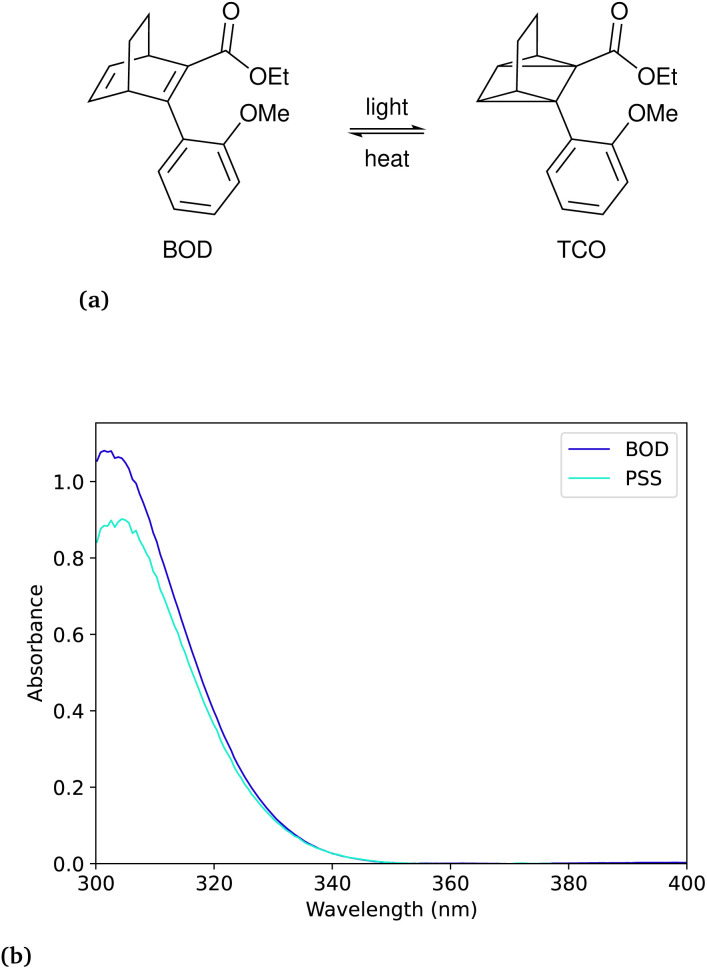
(a) Reaction schematic for the bicyclooctadiene–tetracyclooctane (BOD–TCO) pair. (b) UV-Vis spectrum of the BOD and the photostationary state (PSS) with TCO following irradiation with 308 nm.

The bicyclooctadiene (BOD) we chose to work with was originally published by Quant *et al.*^7^ (2022) and the chemical name for the compound is ethyl-3-(2-methoxyphenyl)bicyclo[2.2.2]octa-2,5-diene-2-carboxylate. In the case of the BOD, we can make an approximation, that the photoproduct will not absorb in the same region as the reactant as reported by Quant *et al.*^7^ In the case of not knowing the absorbance spectrum of the photo-product, one can assume with little conversion that the photo-product will not absorb, to facilitate faster data collection, while also introducing an error. The thermal back-conversion for the photo-product is sufficiently evident at room temperature such that it cannot be neglected. The differential equation for this example is then presented as:7
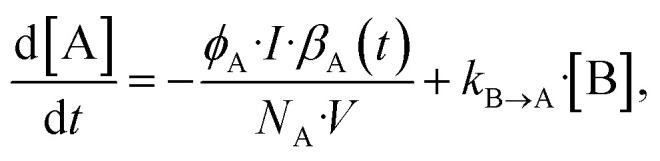
where the photo-isomerisation of the reactant and the thermal back-conversion of the product are included. Since there is no analytical solution to this equation, the problem must be solved numerically, which may be more time-consuming. The difference between using the analytical solution with no thermal back conversion and the numerical solution is shown in Fig. S13 in the ESI.† The optimisation for the best numerical fit is obtained by minimising the squares of the residuals of the fit. This optimisation will usually take a few seconds on a portable PC, which is acceptable (depending on the algorithm and optimisation), but still an order of magnitude slower than the optimisation of the analytical solution, which usually takes less than half a second (Fig. 3 and Table 2).

**Table 2:** The measured quantum yield of the BOD at wavelength 308 nm of irradiation. The measurements were performed at 20 °C in acetonitrile

Irradiation wavelength	Number of measurements	This work	Quant *et al.*^7^ (2022)
308 nm	2	0.146 ± 0.007	0.1443 ± 0.0004

**Fig. 4:**
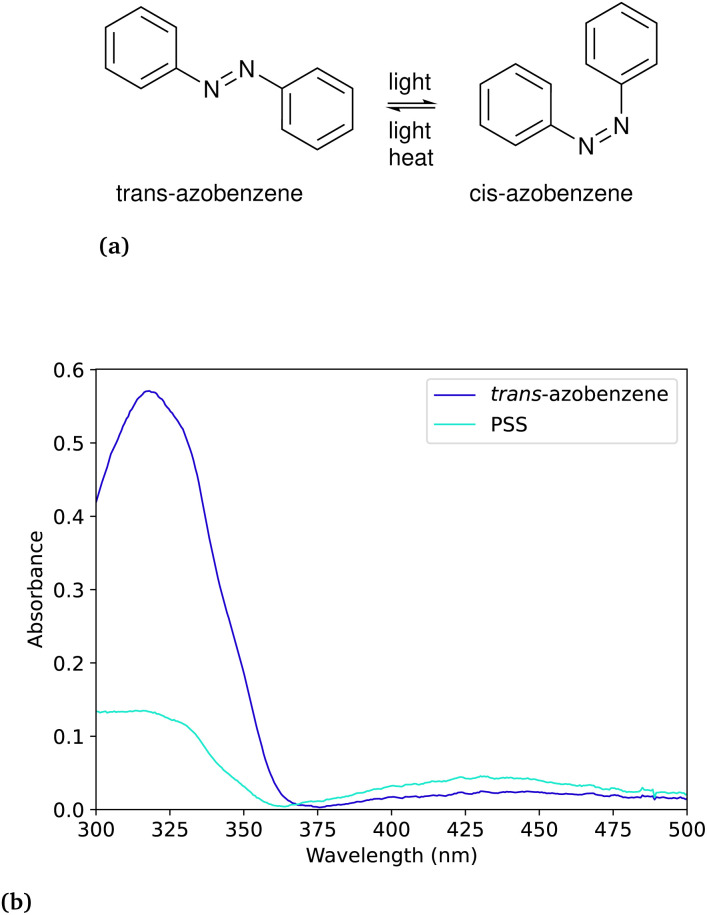
(a) Reaction scheme for the azobenzene photoisomerisation. (b) UV-Vis spectra of the *trans*-azobenzene and the photostationary state (PSS) with *cis*-azobenzene following irradiation at 340 nm.

Additionally, the thermal back-conversion of the BOD was measured at three different temperatures to determine the thermal half-life and thermodynamic parameters. The half-life at room temperature was determined to be 63 seconds, which is in reasonable agreement with the reported 79.8 seconds by Quant *et al.*^7^ The Eyring and Arrhenius plots are shown in Fig. S5† in the ESI (Fig. 4).

### 3.3 Azobenzene

In the case of azobenzene, the compound has a relatively long thermal half-life at room temperature (more than six days) which means that the thermal back-conversion rate can be neglected. The absorbance of the photoproduct *cis*-azobenzene has a relatively insignificant overlap with the absorbance of the reactant *trans*-azobenzene (*ε*(340 nm)_*trans*_ ≈ 12 500 cm^−1^ M^−1^, *ε*(340 nm)_*cis*_ ≈ 200 cm^−1^ M^−1^).^25^ In cases of low conversion percentage, an assumption can be made that the photo-induced back-conversion will not interfere with the concentration change of the reactant. This assumption is made with the purpose of accelerating the data analysis, knowing that it will introduce an error, especially when the product absorbance is unknown. It should be noted that using this method, we do not entirely solve the eqn (1). On the other hand, reaching the photo-stationary state would allow for determining both the forward and backward photoconversion quantum yield. In this work, we will not determine the photoconversion quantum yield of the reverse reaction. In this case, the equation to which the data is fitted can be simplified from eqn (1) to:8
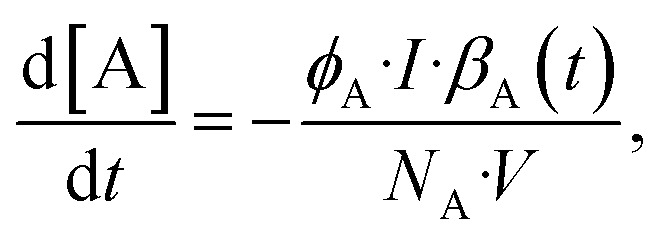
where the terms regarding the reverse photoconversion and the thermal back-conversion is disregarded. In Table 3 we present the values obtained from the experiments and compare to existing reported values.

**Table 3:** The determined quantum yield of the *trans*-to-*cis*-azobenzene photoisomerisation at 340 nm wavelength of irradiation. The measurements were performed at 25 °C in acetonitrile. The reference measurements performed by Ladányi *et al.*^24^ are performed in methanol using an irradiation wavelength of 334 nm

Irradiation wavelength	Number of measurements	This work	Ladányi *et al.*^24^ (2017)
340 nm	3	0.148 ± 0.003	
334 nm	6		0.155 ± 0.006

Additionally, thermal kinetics experiments were performed at several temperatures to determine the thermal half-life. We determined the thermal half-life to be 6.78 days, which is in reasonable agreement with the reported 7.34 days by Vetráková *et al.*^25^ Additionally, the Eyring and Arrhenius plots can be found in Fig. S7, and the thermodynamic values in Table S4 in the ESI.†

## 4 Conclusions

We present here an automated platform for characterising photoisomerisation and thermal back-reaction kinetics for molecular photoswitches such as norbornadienes, bicyclooctadienes, and azobenzenes. In essence, the major time-saving parts include the pre-calibration of the photon fluxes of the LEDs and the automated flow-based sample selection operating in microlitre volumes, allowing for short intermission times between experiments. Additionally, data analysis tools were developed in Python to quickly analyse and visualise the data. Three well-known photoswitch systems were tested, and the results obtained were within the error margins of reported values for the compounds. In combination with the time-saving and material-saving components, the presented platform helps set a new standard for molecular photoswitch characterisation. In conclusion, the newly developed platform allows for accelerated discovery of photoswitch properties in different solvents, which can be used for high-throughput experimentation and screening for optimisation of both molecular structure and environment interactions.

We highlight here the possibility of further automation regarding decision-making, which could be developed to control, for example selection of excitation wavelength, duration of experiment, AI-supported data analysis, and design of experiments (DOE).

## Author contributions

Jacob Lynge Elholm [writing – original draft, data curation, formal analysis, methodology, conceptualisation], Paulius Baronas [writing – review & editing, methodology, conceptualisation], Paul A. Gueben [writing – review & editing, data curation], Victoria Gneiting [writing – review & editing, data curation, formal analysis], Helen Hölzel [writing – review & editing, conceptualisation], Kasper Moth-Poulsen [writing – review & editing, funding acquisition, conceptualisation, supervision].

## Conflicts of interest

There are no conflicts to declare.

## Supplementary Material

DD-004-D5DD00031A-s001

## Data Availability

The data supporting this article has been included as part of the ESI.† The data analysis scripts along with the code for the automated control of the different instruments of this article are available in the Automation folder of the GitHub repository KMP Group at https://www.github.com/Elholm/KMP-Group. Certain required software packages are not supplied as we do not have the licenses to distribute. The file QYEX.py in this repository is based on a Python demo file obtained from Avantes. The data and code presented in the paper are available in a figshare repository at https://doi.org/10.6084/m9.figshare.28254350.

## References

[cit1] Salthouse R. J., Moth-Poulsen K. (2024). J. Mater. Chem. A.

[cit2] van Leeuwen T., Lubbe A. S., Štacko P., Wezenberg S. J., Feringa B. L. (2017). Nat. Rev. Chem..

[cit3] Irie M. (2000). Chem. Rev..

[cit4] Hillers-Bendtsen A. E., Elholm J. L., Obel O. B., Hölzel H., Moth-Poulsen K., Mikkelsen K. V. (2023). Angew. Chem., Int. Ed..

[cit5] Orrego-Hernández J., Dreos A., Moth-Poulsen K. (2020). Acc. Chem. Res..

[cit6] Quant M., Lennartson A., Dreos A., Kuisma M., Erhart P., Börjesson K., Moth-Poulsen K. (2016). Chem.–Eur. J..

[cit7] Quant M., Hillers-Bendtsen A. E., Ghasemi S., Erdelyi M., Wang Z., Muhammad L. M., Kann N., Mikkelsen K. V., Moth-Poulsen K. (2022). Chem. Sci..

[cit8] Hillers-Bendtsen A. E., Quant M., Moth-Poulsen K., Mikkelsen K. V. (2021). J. Mater. Chem. A.

[cit9] Elholm J. L., Liasi Z., Mikkelsen M. K., Hillers-Bendtsen A. E., Mikkelsen K. V. (2023). Phys. Chem. Chem. Phys..

[cit10] Aleotti F., Petropoulos V., Van Overeem H., Pettini M., Mancinelli M., Pecorari D., Maiuri M., Medri R., Mazzanti A., Preda F., Perri A., Polli D., Conti I., Cerullo G., Garavelli M. (2023). J. Mater. Chem. A.

[cit11] Siewertsen R., Neumann H., Buchheim-Stehn B., Herges R., Näther C., Renth F., Temps F. (2009). J. Am. Chem. Soc..

[cit12] Wang Z., Moïse H., Cacciarini M., Nielsen M. B., Morikawa M.-a., Kimizuka N., Moth-Poulsen K. (2021). Adv. Sci..

[cit13] Börjesson K., Lennartson A., Moth-Poulsen K. (2013). ACS Sustain. Chem. Eng..

[cit14] Volker A., Steen J. D., Crespi S. (2024). Beilstein J. Org. Chem..

[cit15] Megerle U., Lechner R., König B., Riedle E. (2010). Photochem. Photobiol. Sci..

[cit16] Kumbhakar M., Khandelwal A., Jha S. K., Kante M. V., Keßler P., Lemmer U., Hahn H., Aghassi-Hagmann J., Colsmann A., Breitung B., Velasco L., Schweidler S. (2023). Adv. Energy Mater..

[cit17] Griffiths R.-R., Greenfield J. L., Thawani A. R., Jamasb A. R., Moss H. B., Bourached A., Jones P., McCorkindale W., Aldrick A. A., Fuchter M. J., Lee A. A. (2022). Chem. Sci..

[cit18] Jevric M., Petersen A. U., Mansø M., Kumar Singh S., Wang Z., Dreos A., Sumby C., Nielsen M. B., Börjesson K., Erhart P., Moth-Poulsen K. (2018). Chem.–Eur. J..

[cit19] Volfova H. , Hu Q. and Riedle E., EPA Newsletter, 2019, pp. 51–69

[cit20] Hatchard C. G., Parker C. A., Bowen E. J. (1956). Proc. R. Soc. London, Ser. A.

[cit21] Lehóczki T., Józsa É., Ősz K. (2013). J. Photochem. Photobiol., A.

[cit22] Bowman W. D., Demas J. N. (1976). J. Phys. Chem..

[cit23] Stadler E., Eibel A., Fast D., Freißmuth H., Holly C., Wiech M., Moszner N., Gescheidt G. (2018). Photochem. Photobiol. Sci..

[cit24] Ladányi V., Dvořák P., Al Anshori J., Vetráková Ľ., Wirz J., Heger D. (2017). Photochem. Photobiol. Sci..

[cit25] Vetráková Ľ., Ladányi V., Al Anshori J., Dvořák P., Wirz J., Heger D. (2017). Photochem. Photobiol. Sci..

