# Supplementary material for: An automated photo-isomerisation and kinetics characterisation system for molecular photoswitches
Source: Digit Discov. 2025 Jun 30;4(8):2045–51. doi: 10.1039/d5dd00031a (PMC12232385; doi:10.1039/d5dd00031a)
Supplement: DD-004-D5DD00031A-s001 [file DD-004-D5DD00031A-s001.pdf]

Supplementary Information (SI) for Digital Discovery.
This journal is © The Royal Society of Chemistry 2025

An automated photo-isomerisation and kinetics characterisation setup for molecular photoswitches: Supporting information

Jacob Lynge Elholm, Paulius Baronas, Paul A. Gueben, Victoria Gneiting, Helen Hölzel, and Kasper Moth-Poulsen*

Department of Chemical Engineering, Universitat Politècnica de Catalunya, EEBE, Eduard Maristany 10-14, 08019 Barcelona, Spain

E-mail: kasper.moth.poulsen@upc.edu

Contents

1 Kinetics studies **5**

1.1 Norbornadiene 5

1.2 Bicyclooctadiene 8

1.3 Azobenzene 10

2 Quantum yield measurements **12**

2.1 Norbornadiene 12

2.2 Bicyclooctadiene 14

2.3 Azobenzene 15

2.4 LED photon flux determination 17

3 Synthesis routes **18**

3.1 Norbornadiene 18

3.2 Bicyclooctadiene 19

4 Setup details **20**

4.1 Hardware details 20

4.2 Optimised workflow 22

4.3 Graphical user interface 23

4.4 User manuals 23

List of Figures

S1 The data included to calculate the k value at a temperature of 70 ºC. 5

S2 Thermal conversion from QC to NBD. Recorded at three different temperatures in toluene. 6

S3 Fitted plots for the kinetics of the Norbornadiene. 7
S4 Thermal conversion from the PSS of conversion (mix of TCO and BOD) to BOD. Recorded at four different temperatures in acetonitrile. 8
S5 Graphs fitted to the determined rate constants follow the Arrhenius and the Eyring equation. 9
S6 Thermal conversion from the PSS of conversion (mix of *cis*-azobenzene and *trans*-azobenzene) to *trans*-azobenzene. Recorded at three different temperatures in toluene. 10
S7 Fitted Arrhenius and Eyring plots for the kinetics of the azobenzene. 11
S8 Norbornadiene-quadricyclane pair UV-Vis spectra and the emission spectrum of the 340 nm LED to showcase the spectral overlap. 12
S9 The difference between the quantum yield fit with and without thermal back reaction rate at 25 ºC. The obtained quantum yield for the fit without thermal back reaction rate was 0.6726 and with thermal back reaction rate 0.6790 was obtained. 12
S10 The conversion of the NBD with only irradiation from the UV lamp. In roughly 10 minutes of irradiation the compound has converted around 10%. 13
S11 Examples for the NBD quantum yield analyses. 13
S12 UV-Vis spectra of the BOD and PSS with a mix of BOD and TCO. 14
S13 Examples of the quantum yield fit with and without the thermal back conversion term. 14
S14 UV-Vis spectra of the *trans*-azobenzene and the PSS with a mix of *trans*-azobenzene and *cis*-azobenzene 15
S15 Examples of quantum yield fit for the azobenzene. 16
S16 The normalised intensities of the LED array. 17
S17 1H-NMR (400 MHz, Chloroform-d, room temperature) spectrum of the NBD. 18
S18 Synthesis route for the BOD. 19

S19 1H-NMR (400 MHz, Chloroform-d, room temperature) spectrum of the BOD. 20
S20 Picture of the physical setup in our lab. 21
S21 Schematic of the optimised workflow. The yellow outlined boxes contain manual labour, the green outlined boxes contain semi-automatic computerised tasks, and the green boxes contain fully automated tasks. 22
S22 Snapshot of the graphical user interface developed in Python for the automation of the equipment described in the setup. 23

List of Tables

S1 The determined rate constants at the different experiment durations. 6
S2 Thermodynamic values for the NBD 7
S3 Thermodynamic values for the bicyclooctadiene. 9
S4 Thermodynamic values for the azobenzene. 11

1 Kinetics studies

1.1 Norbornadiene

Since Norbornadiene is the slowest to back convert, we show here the significance of letting the sample fully back convert before stopping the experiment. For one experiment the values of the rate constant, k, was evaluated using different parts of the time evolution to simulate an experiment performed in shorter time. The effects of letting the compound fully convert on the rate constant do not seem consistent as including more points in the beginning causes the rate constant to increase and including later points causes the rate constant to decrease again. With a 10% difference between the determined rate constant between long and short experiments, this benchmark demonstrates the potential efficiency increase in performing experiments for shorter time with a small decrease in accuracy.

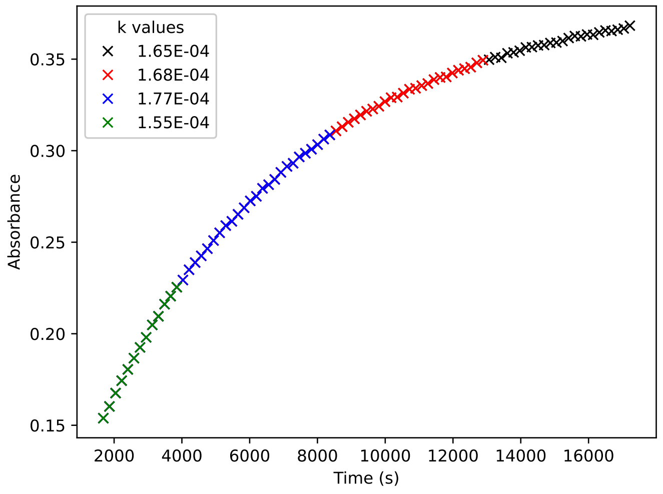

Figure S1: The data included to calculate the k value at a temperature of 70 ºC.

Table S1: The determined rate constants at the different experiment durations.

Duration of experiment included (s)	Determined rate constant (1/s)
2000	1.65e-4
5000	1.68e-4
10000	1.77e-4
15000	1.55e-4

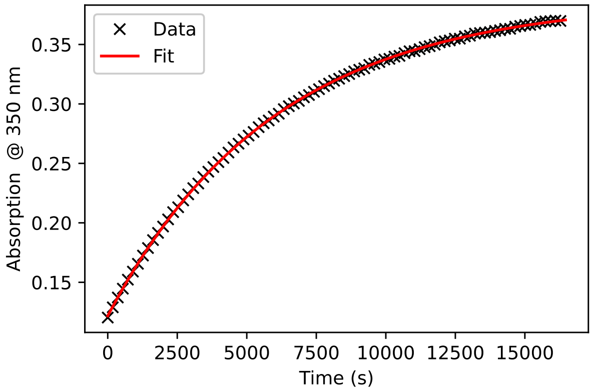

(a) 70 ºC. K = 1.6534e-04

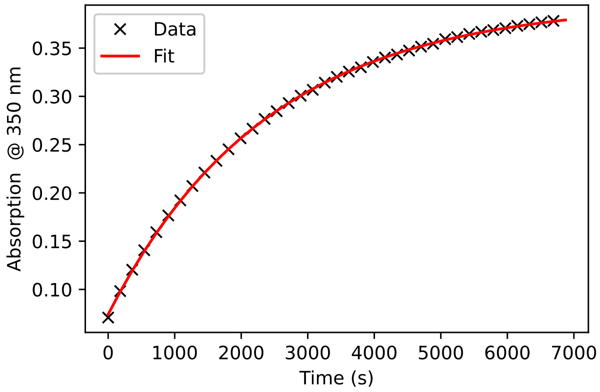

(b) 80 ºC. K = 4.1711e-04

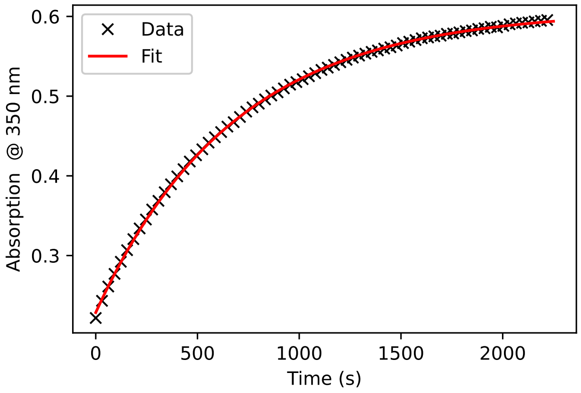

(c) 90 ºC. K = 1.6196e-03

Figure S2: Thermal conversion from QC to NBD. Recorded at three different temperatures in toluene.

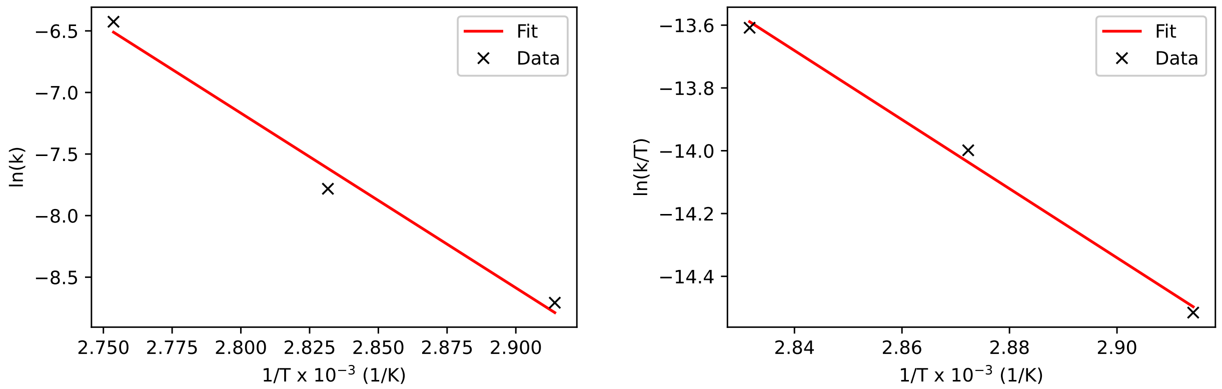

(a) Arrhenius plot

(b) Eyring plot

Figure S3: Fitted plots for the kinetics of the Norbornadiene.

Table S2: Thermodynamic values for the NBD

Property	Value
$t_{1/2}$ (25ºC)	27 days
ΔH	115.039935 kJ/mol
ΔS	16.1 J/mol K
ΔG (25ºC)	117.974 kJ/mol

1.2 Bicyclooctadiene

The chemical name of the compound is ethyl-3-(2-methoxyphenyl)bicyclo[2.2.2]octa-2,5-diene-2-carboxylate. The absorption of the BOD was measured at 310 nm. The fitted function is an exponential function.

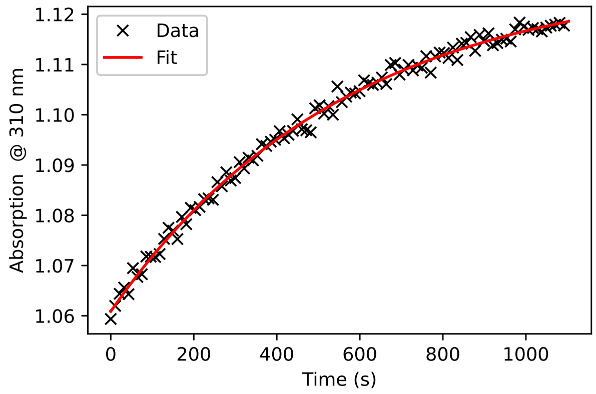

(a) 10 ºC. k = 1.7928e-03

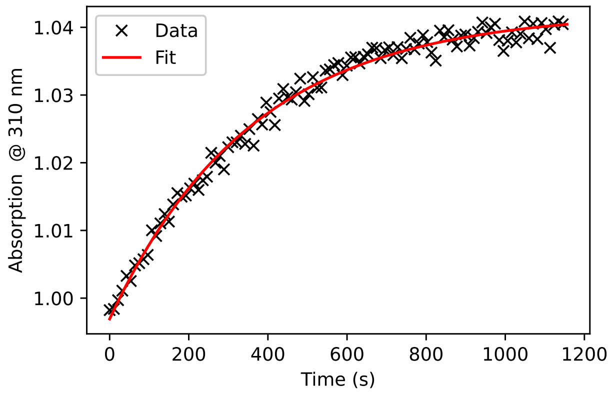

(b) 15 ºC. k = 2.7024e-03

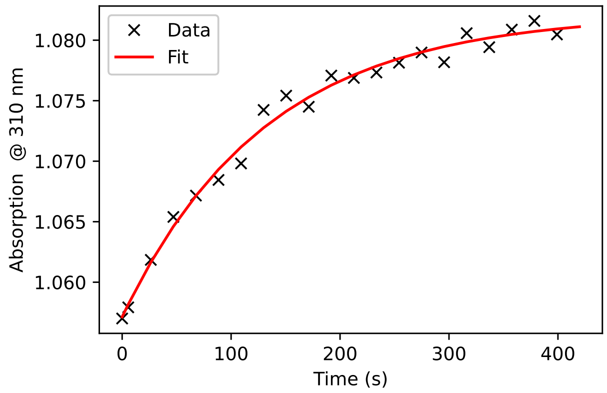

(c) 20 ºC. k = 5.5497e-03

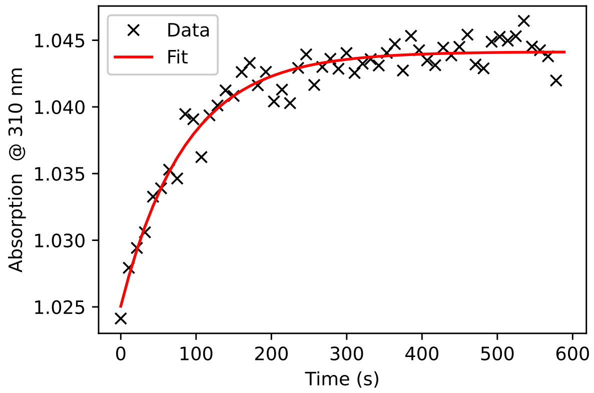

(d) 25 ºC. k = 1.2076e-02

Figure S4: Thermal conversion from the PSS of conversion (mix of TCO and BOD) to BOD. Recorded at four different temperatures in acetonitrile.

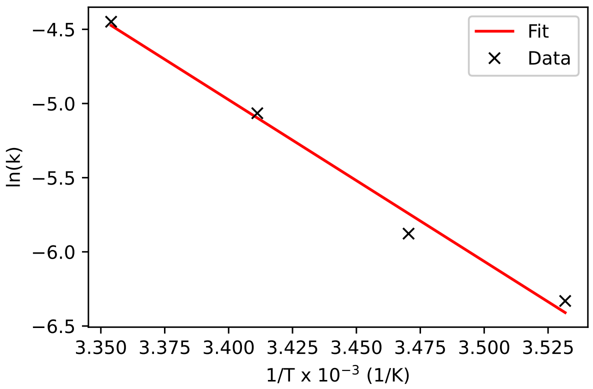

(a) Arrhenius plot

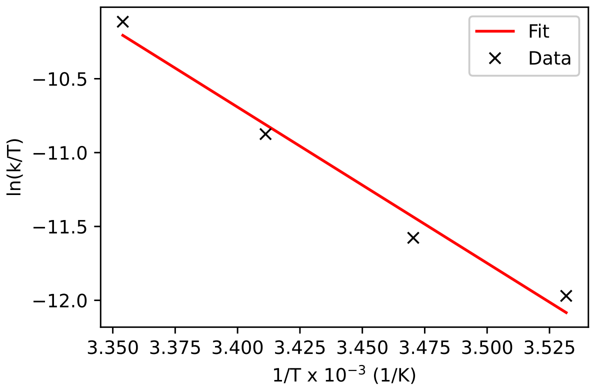

(b) Eyring plot

Figure S5: Graphs fitted to the determined rate constants follow the Arrhenius and the Eyring equation.

Table S3: Thermodynamic values for the bicyclooctadiene.

Property	Value
$t_{1/2}$ (25ºC)	63 s
A	7.10e+13 1/s
ΔH	87.824995 kJ/mol
ΔS	12.2 J/mol
ΔG (25ºC)	90.240 kJ/mol

1.3 Azobenzene

The chemical name of the azobenzene is (E)-diphenyldiazene.

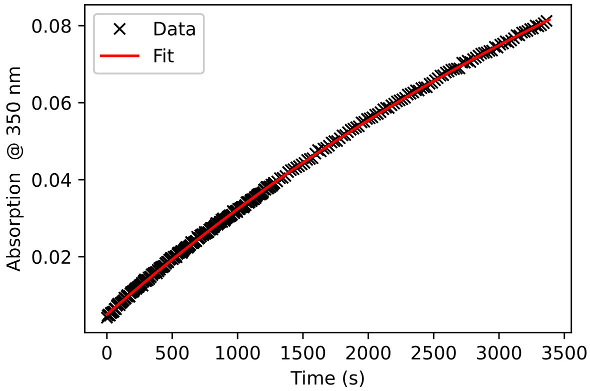

(a) 70 ºC. k = 1.7041e-04

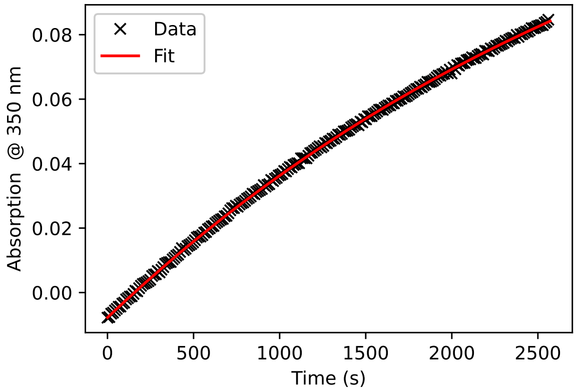

(b) 75 ºC. k = 2.8992e-04

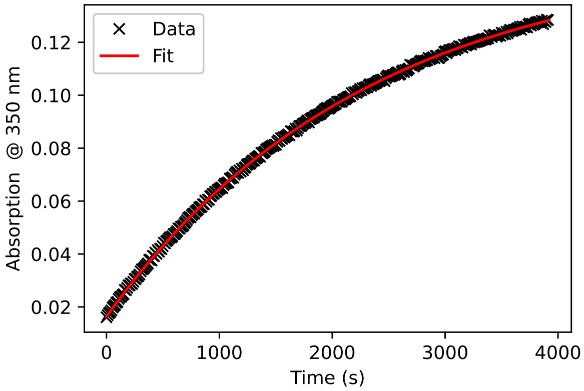

(c) 80 ºC. k = 4.3443e-04

Figure S6: Thermal conversion from the PSS of conversion (mix of *cis*-azobenzene and *trans*-azobenzene) to *trans*-azobenzene. Recorded at three different temperatures in toluene.

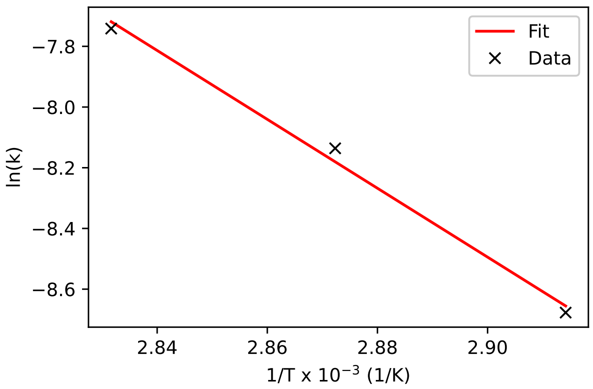

(a) Arrhenius plot

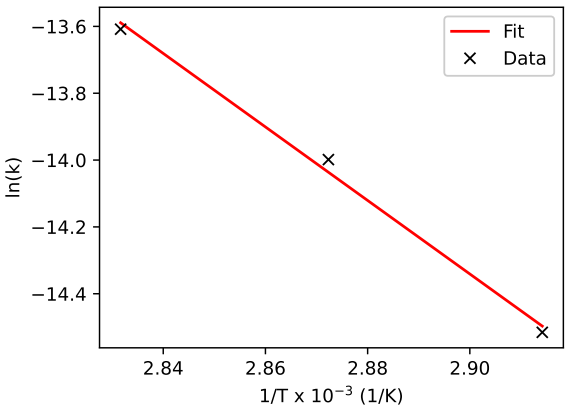

(b) Eyring plot

Figure S7: Fitted Arrhenius and Eyring plots for the kinetics of the azobenzene.

Table S4: Thermodynamic values for the azobenzene.

Property	Value
$t_{1/2}$ (25ºC)	6.796 days
A	3.99e+10 1/s
ΔH	91.452630 kJ/mol
ΔS	-51.6 J/mol K
ΔG (25ºC)	94.347 kJ/mol

2 Quantum yield measurements

2.1 Norbornadiene

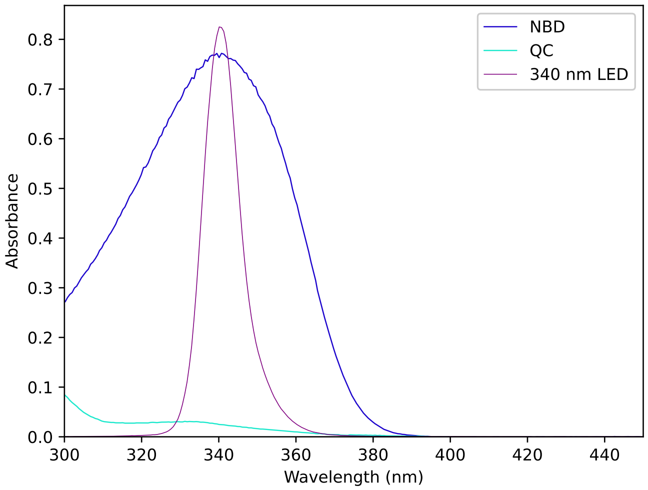

Figure S8: Norbornadiene-quadricyclane pair UV-Vis spectra and the emission spectrum of the 340 nm LED to showcase the spectral overlap.

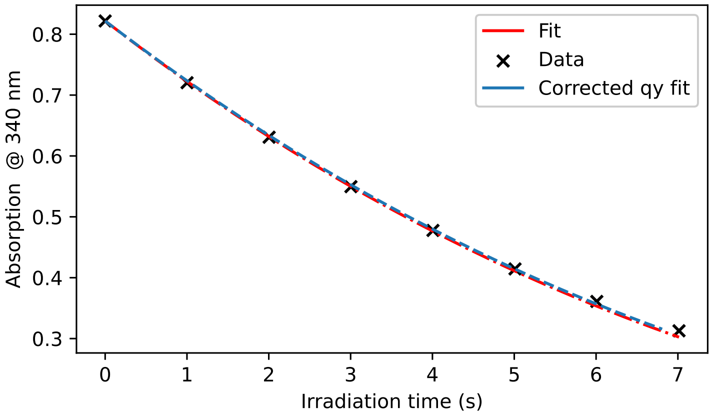

Figure S9: The difference between the quantum yield fit with and without thermal back reaction rate at 25 ºC. The obtained quantum yield for the fit without thermal back reaction rate was 0.6726 and with thermal back reaction rate 0.6790 was obtained.

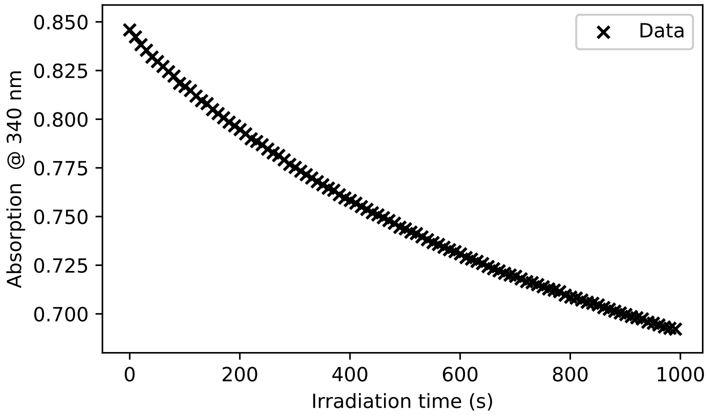

Figure S10: The conversion of the NBD with only irradiation from the UV lamp. In roughly 10 minutes of irradiation the compound has converted around 10%.

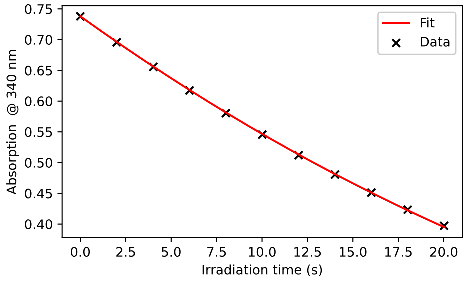

(a) qy = 0.701

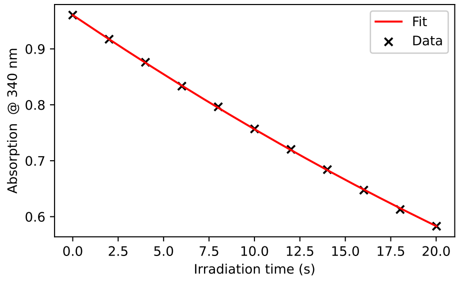

(b) qy = 0.683

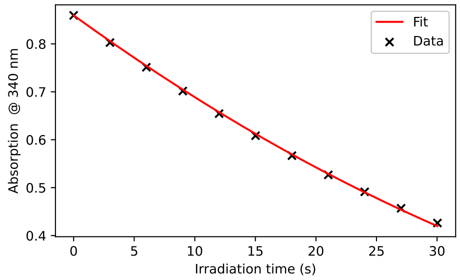

(c) qy = 0.697

Figure S11: Examples for the NBD quantum yield analyses.

2.2 Bicyclooctadiene

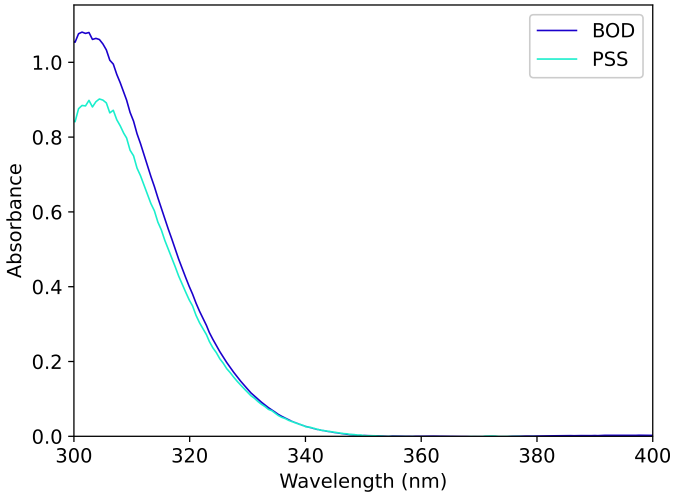

Figure S12: UV-Vis spectra of the BOD and PSS with a mix of BOD and TCO.

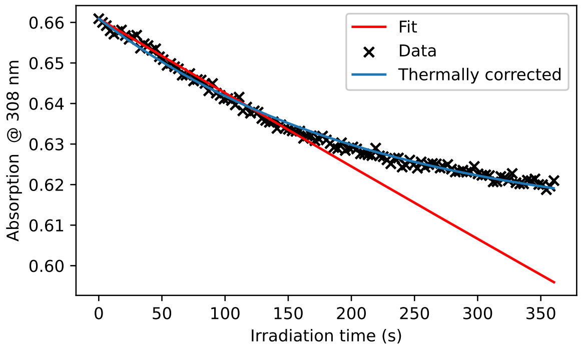

(a) qy = 14.4 %

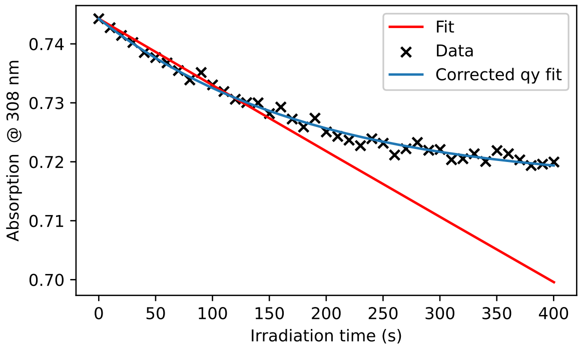

(b) qy = 15.2110 %

Figure S13: Examples of the quantum yield fit with and without the thermal back conversion term.

2.3 Azobenzene

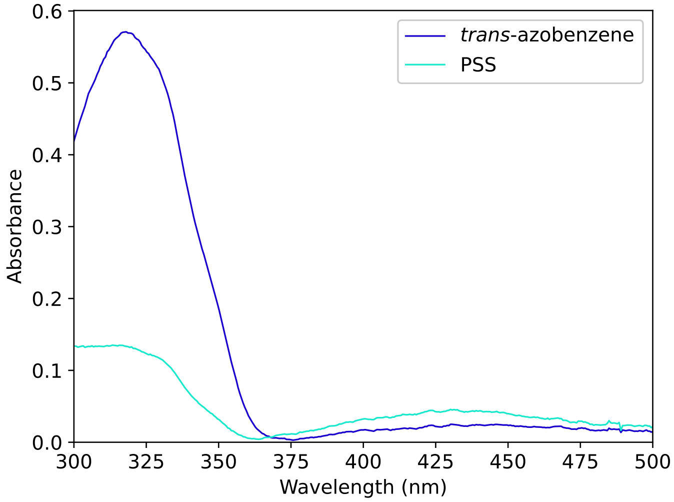

Figure S14: UV-Vis spectra of the *trans*-azobenzene and the PSS with a mix of *trans*-azobenzene and *cis*-azobenzene

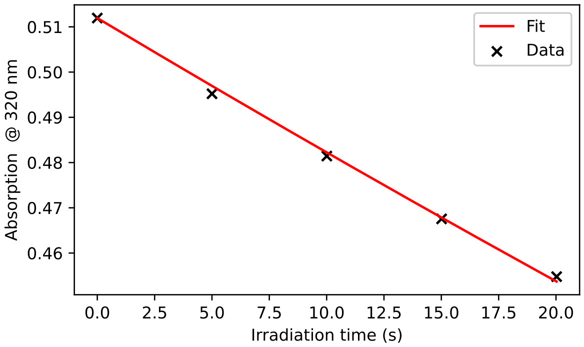

(a) QY = 0.1444

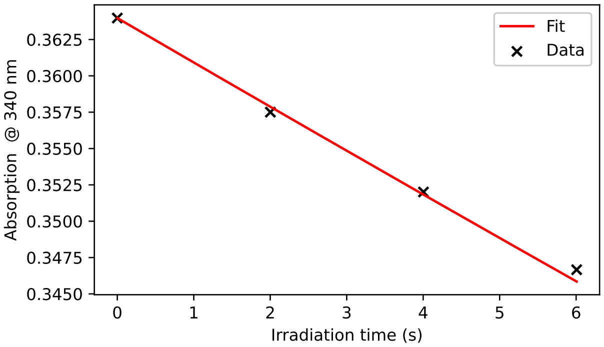

(b) QY = 0.1499

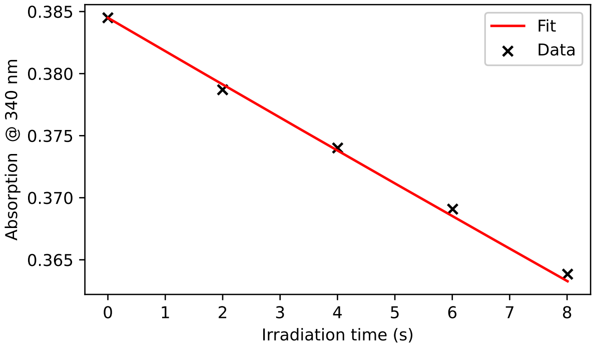

(c) QY = 0.1501

Figure S15: Examples of quantum yield fit for the azobenzene.

2.4 LED photon flux determination

The LED photon flux determination was performed with a powermeter from Thorlabs consisting of a photodiode power sensor S120VC connected to a PM100USB power and energy meter interface with USB operation that handles the data transfer from the power sensor to the computer. The photodiode power sensor was placed after the cuvette, such that the cuvette is between the light source and the power sensor, and inside the cuvette holder to measure the power coming through from the LEDs. In this case, only the photons that pass through the cuvette are measured by the power sensor. This is done for two reasons, one being that the window of the cuvette is smaller than the irradiation beam, so if the power was measured before the cuvette, photons that would not enter the sample chamber would still be included in the power measurement. The second reason is that the reflection of the quartz cuvette would not be accounted for if the power was measured before the cuvette, but that factor is included if the measurement of photons takes place after passing through the cuvette.

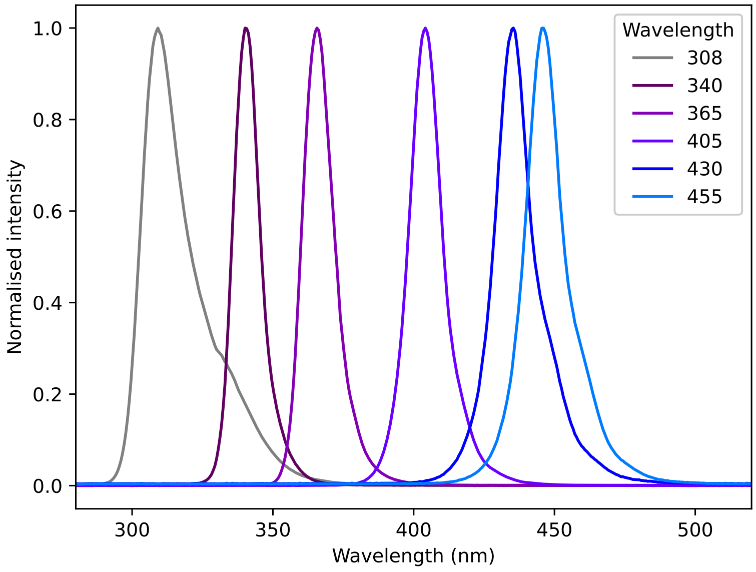

Figure S16: The normalised intensities of the LED array.

3 Synthesis routes

3.1 Norbornadiene

The norbornadiene was synthesised according to the synthesis route for NBD-2h presented by Quant et al.[1] The chemical name of the compound is 2-cyano-3-(3,4-dimethoxyphenyl)norbornadiene.

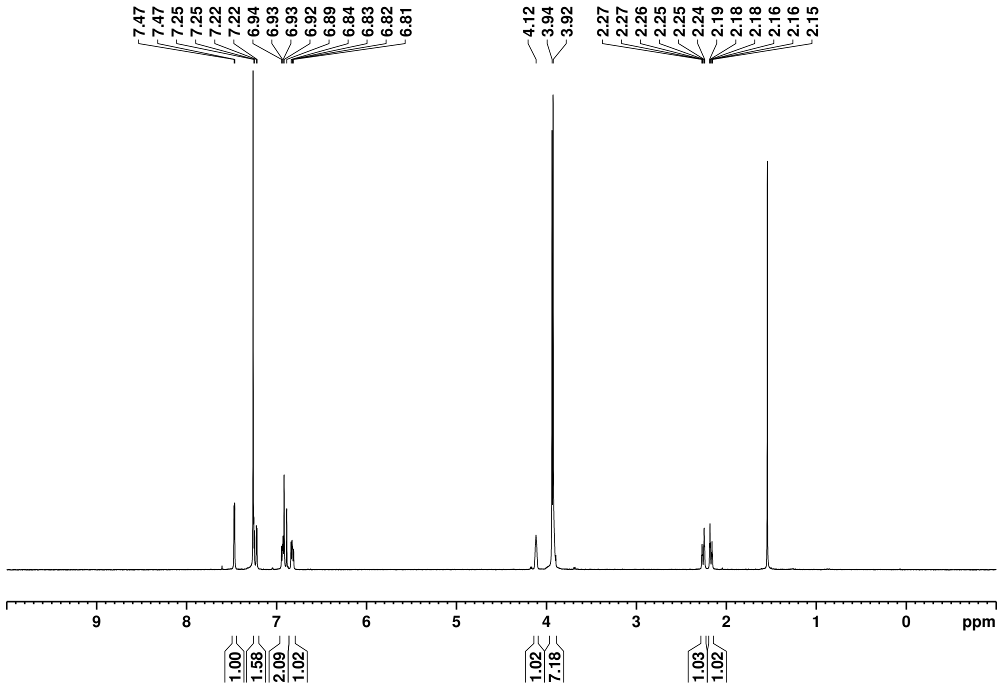

Figure S17: 1H-NMR (400 MHz, Chloroform-d, room temperature) spectrum of the NBD.

3.2 Bicyclooctadiene

The synthesis of the bicyclooctadiene (BOD) was carried out in three steps following the procedure described by Quant et al.[2]

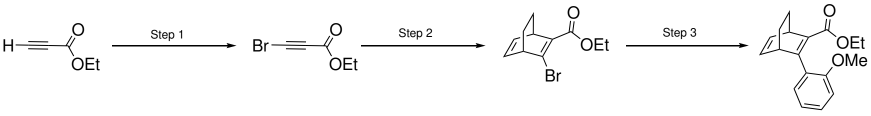

Figure S18: Synthesis route for the BOD.

Step 1: In a degassed round bottomed flask containing the solvent (acetone, 15mL), ethyl propiolate (2.55 mmol, 1 equiv.), NBS (2.8 mmol, 1.1 equiv.) and AgNO3 (0.25 mmol, 0.1 equiv.) are added. The mixture is stirred at room temperature for 3 hours covered from sunlight with aluminium foil. After 3 hours, the mixture is extracted with DCM and water and the organic layers are gathered. Rotatory evaporation of the solvent afforded the desired product with a yield of 98%. Step 2: The Diels-Alder reaction was performed using the same procedure showed in the paper. The purified product was obtained with a yield of 44%. Step 3: The Suzuki cross-coupling between the product from step 2 and 2-methoxyphenyl boronic acid was performed using the same procedure showed in the paper. The purified product was obtained with a yield of 38%.

1H NMR (400 MHz, Chloroform-d) 7.27 (m, 1H, Ar-H), 6.98 (dd, J = 7.7, 1.8 Hz, 1H, Ar-H), 6.92 – 6.87 (m, 2H, Ar-H), 6.47 (ddd, J = 7.5, 6.1, 1.6 Hz, 1H, HRC=CRH), 6.41 (ddd, J = 7.4, 5.8, 1.7 Hz, 1H, HRC=CRH), 4.28 – 4.24 (m, 1H, HR3C), 4.00 – 3.91 (m, 2H, COOCH2CH3), 3.80 (s, 3H, ROCH3), 3.74 (m, 1H, HR3C), 1.59 (m, 2H, RH2C-CH2R), 1.40 (m, 2H, RH2C-CH2R), 0.93 (t, J = 7.1 Hz, 3H, COOCH2CH3).

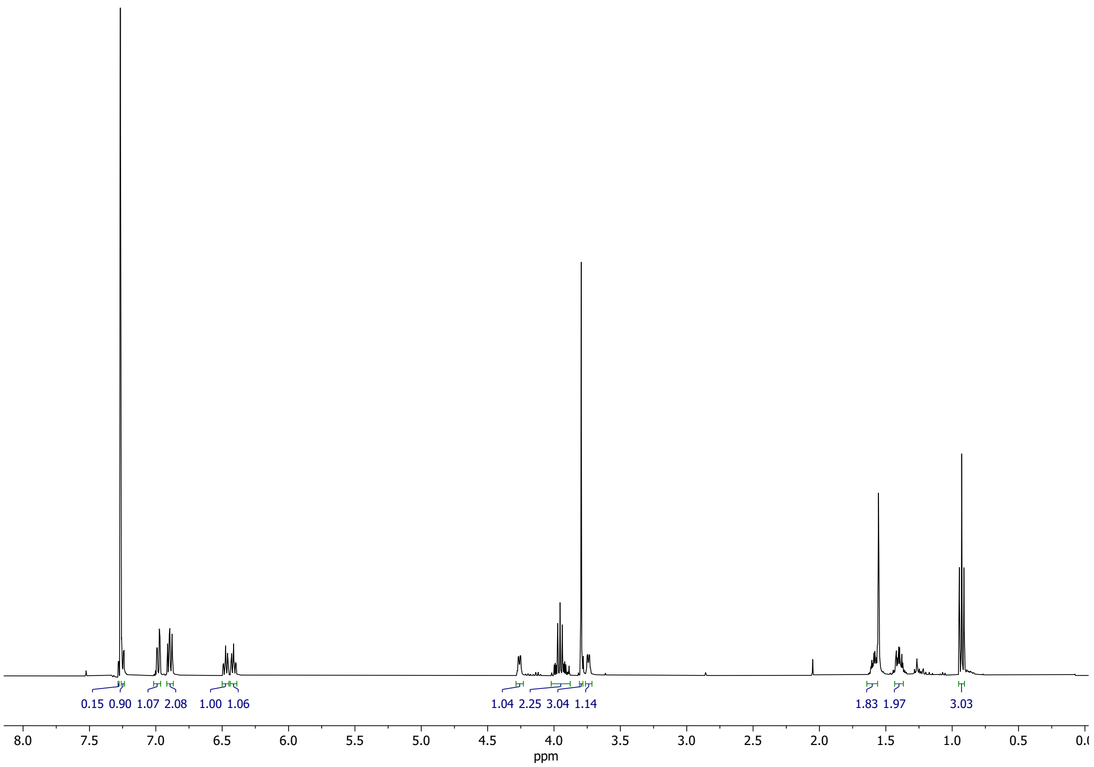

Figure S19: 1H-NMR (400 MHz, Chloroform-d, room temperature) spectrum of the BOD.

4 Setup details

4.1 Hardware details

List of instruments in the setup each marked with the corresponding number in Figure S20.

1. Portable computer with software installed
2. Temperature-controlled cuvette holder (Quantum Northwest qPod 3e)
3. UV-Lamp (Avantes AvaLight-D(H)-S)
4. Spectrometer (Avantes AvaSpec-ULS2048CL-EVO)
5. Valve (Knauer Valve AVQ63AF, Valve unifier AWA01XA)
6. Pump (Vapourtec SF10 Pump)
7. LED array (Thorlabs Fiber-Coupled LEDs; M280F5, M310F1,,M340F4, M365FP1, M405F3, M430F1, M455F3)
8. Sample rack (In-house design and 3D-print)

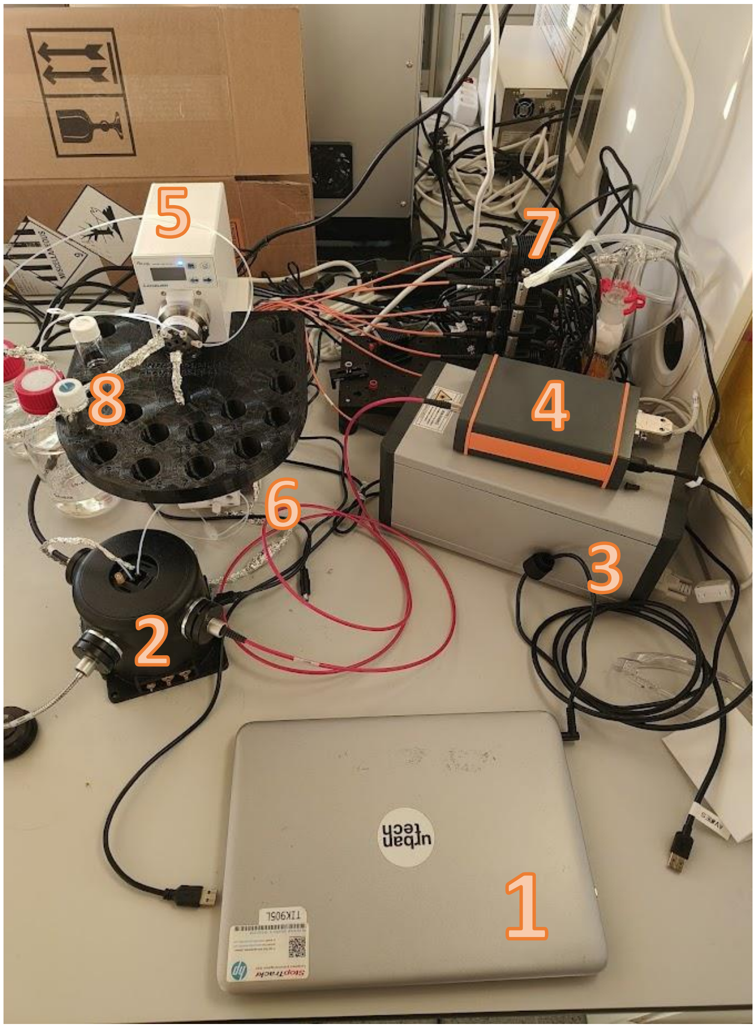

Figure S20: Picture of the physical setup in our lab.

9. Flow cuvette (Starna Cells Type 583.4.2F-Q-10/Z15) (inside the cuvette holder)

The optical fiber connecting the LEDs and the lamp to the cuvette holder is a 1-to-7 Fan-Out Fiber Optic Bundle (BF76HS01) from Thorlabs.

The Python code and program is hosted on GitHub on the repository of KMP-Group https://github.com/Elholm/KMP-Group. Additionally to control the hardware, some proprietary APIs are necessary. To control the spectrometer and the lamp shutter from Avantes, it is required to obtain the API from them directly. To control the LEDs and the powermeter, an API is downloadable from the Thorlabs website. To control the powermeter the program Optical Power Monitor is required. To control the LEDs the program upSERIES©

is required.

The wavelengths of the LEDs were chosen based on the absorption wavelengths of the photoswitches that we mostly work with. In the setup, an LED can easily be swapped for another desired LED. The LEDs available from Thorlabs range from 280 nm to 1450 nm.

4.2 Optimised workflow

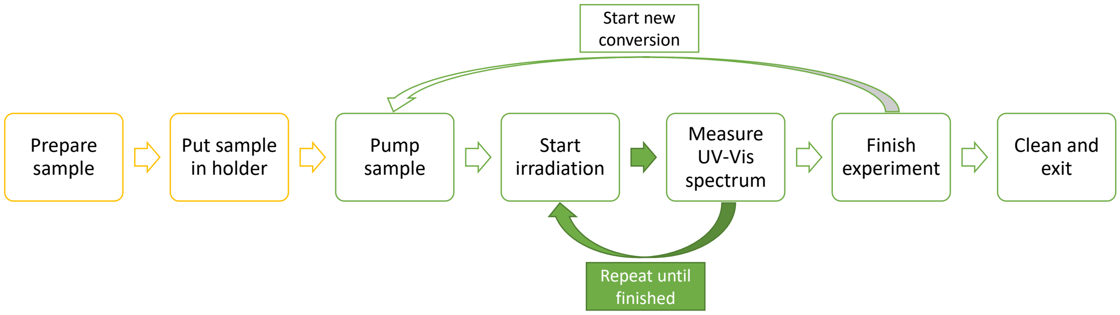

Figure S21: Schematic of the optimised workflow. The yellow outlined boxes contain manual labour, the green outlined boxes contain semi-automatic computerised tasks, and the green boxes contain fully automated tasks.

4.3 Graphical user interface

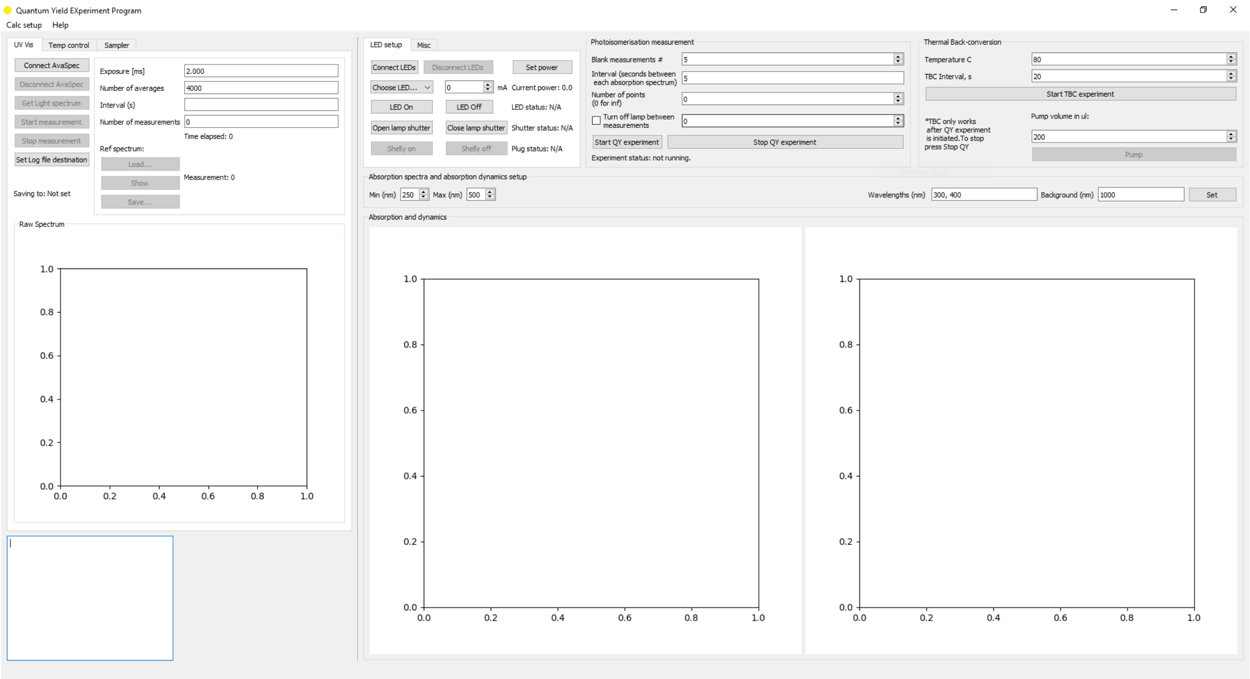

Figure S22: Snapshot of the graphical user interface developed in Python for the automation of the equipment described in the setup.

4.4 User manuals

Quantum Yield Analysis (QYA)

Load the file. Fill in either the “Concentration” or the “Extinction coefficient.” Select either “Photon flux” or the “Quantum yield”, depending on which one of the two you know. In the case of knowing the photon flux: “Load LED”, and enter the wavelength of the LED, its power, as well as the cuvette size (either the 80uL flow cell, or the normal one). In doing so, the sample volume, the cuvette path length, and the LED wavelength will be set automatically. In the case of knowing the quantum yield, the sample volume, the cuvette path length, and the LED wavelength must be input manually. “Zero-point wavelength”: the point where there is no absorption. “Number of points for fit”: usually 15. “Analysis wavelength”: the maximum absorption to see the time evolution. Press “Save params”. Set

the “Starting time”, which is the starting point (equal to the number of blank measurements from the loaded file).

Quantum Yield EXperiment (QYEX)

QYA program instructions

Quantum Yield EXperiment (QYEX) Program: Instructions

Press the start button to open „Quantum Yield Experiment Program“. Connect both USBs to the laptop. Go to “UV-Vis” window. Select “Connect AvaSpec” to connect the spectrometer. Turn on the deuterium lamp (UV). Set “Number of measurements” to 1. a) Normal cuvette: Set “Exposure [ms]” to 0.8, and “Number of averages” to 100. b) Flow cuvette: Set “Exposure [ms]” to 2.0, and “Number of averages” to 100. Pump 2 mL of the solvent to fill the cuvette completely and take the light spectrum. Press “Get Light spectrum” to get the light spectrum (or reference spectrum) of the clean sample, or press “Load” to select an already saved light spectrum. To get 1 absorbance spectrum: Press “Open lamp shutter.” Press “Start measurement.” Press “Close lamp shutter.” Save the light spectrum. (Note: you can also load an already existing light spectrum) Click on “Connect LEDs.” Go to “Select LED” and choose the correct one. Set the power for the chosen LED, maximum is listed here in mA: 308nm = 600 340nm = 600 365nm = 1200 405nm = 1200 Press “Set power.” Set the number of “Blank measurements” (usually 5) and the “Interval” (usually 1). To change the temperature: Open “T’App” on the desktop. Connect COM12. Go to “Change target” to change the temperature and click “On.” Note: turn on the stirrer when using the normal cuvette; leave the stirrer turned off when using the flow cuvette. Press “Start QY experiment.” Once the system stabilizes again, start back conversion by pressing “Start TBC experiment.” (Note: repeat step 13 to change the temperature for the backward reaction when necessary). Select “Stop QY experiment” once the back conversion is done.

References

(1) Quant, M.; Lennartson, A.; Dreos, A.; Kuisma, M.; Erhart, P.; Börjesson, K.; Moth-Poulsen, K. Low Molecular Weight Norbornadiene Derivatives for Molecular Solar-Thermal Energy Storage. *Chemistry A European Journal* **2016**, *22*, 13265–13274.

(2) Quant, M.; Hillers-Bendtsen, A. E.; Ghasemi, S.; Erdelyi, M.; Wang, Z.; Muhammad, L. M.; Kann, N.; Mikkelsen, K. V.; Moth-Poulsen, K. Synthesis, Characterization and Computational Evaluation of Bicyclooctadienes towards Molecular Solar Thermal Energy Storage. *Chemical Science* **2022**, *13*, 834–841.
